# Location of Reentry Tears Affects False Lumen Thrombosis in Aortic Dissection Following TEVAR

**DOI:** 10.1177/1526602820917962

**Published:** 2020-05-04

**Authors:** Chlöe Harriet Armour, Claudia Menichini, Kristijonas Milinis, Richard G. J. Gibbs, Xiao Yun Xu

**Affiliations:** 1 Department of Chemical Engineering, Imperial College London, UK; 2 Regional Vascular Unit, St Mary’s Hospital, Imperial College Healthcare NHS Trust, London, UK

**Keywords:** aortic dissection, computational fluid dynamics, endovascular procedures, false lumen, patient-specific geometrical models, reentry tear, stent-graft, thrombosis, thoracic endovascular aortic repair

## Abstract

**Purpose:** To report a study that assesses the influence of the distance between the distal end of a thoracic stent-graft and the first reentry tear (SG-FRT) on the progression of false lumen (FL) thrombosis in patients who underwent thoracic endovascular aortic repair (TEVAR). **Materials and Methods:** Three patient-specific geometrical models were reconstructed from postoperative computed tomography scans. Two additional models were created by artificially changing the SG-FRT distance in patients 1 and 2. In all 5 models, computational fluid dynamics simulations coupled with thrombus formation modeling were performed at physiological flow conditions. Predicted FL thrombosis was compared to follow-up scans. **Results:** There was reduced false lumen flow and low time-averaged wall shear stress (TAWSS) in patients with large SG-FRT distances. Predicted thrombus formation and growth were consistent with follow-up scans for all patients. Reducing the SG-FRT distance by 30 mm in patient 1 increased the flow and time-averaged wall shear stress in the upper abdominal FL, reducing the thrombus volume by 9.6%. Increasing the SG-FRT distance in patient 2 resulted in faster thoracic thrombosis and increased total thrombus volume. **Conclusion:** The location of reentry tears can influence the progression of FL thrombosis following TEVAR. The more distal the reentry tear in the aorta the more likely it is that FL thrombosis will occur. Hence, the distal landing zone of the stent-graft should be chosen carefully to ensure a sufficient SG-FRT distance.

## Introduction

Aortic dissection affects 3 to 5 of 100,000 individuals every year,^1^ and approximately one-third of dissections are type B. It has been reported that roughly 90% of patients with uncomplicated type B dissections will survive to hospital discharge if treated with medical therapy alone.^2^ Although thoracic endovascular aortic repair (TEVAR) has emerged as a viable option in the treatment of type B dissection, its use was mainly limited to complicated cases to replace high-risk open surgeries.^2^ However, recent studies have highlighted the efficacy of TEVAR in uncomplicated cases, with a significantly higher number of patients achieving the desired complete thrombosis of the false lumen (FL) following TEVAR.^3–6^

While there is evidence suggesting that TEVAR is an effective treatment method for uncomplicated cases, it is still an invasive procedure in which a stent-graft is inserted into the true lumen (TL), covering the primary entry tear to reduce blood flow to the FL and promote thrombosis. This procedure carries risks, which include standard surgical procedural risks and late-onset risks induced by the stent-graft, such as new reentry tears, endoleaks, retrograde dissection, and stent-graft migration, all of which may require further intervention.^7,8^ With the potential for such complications, it is desirable to understand and be able to predict the progression of the disease after TEVAR. This will allow, first, patients to be selected for TEVAR only if there is a high chance of FL thrombosis, and second, clinicians to tailor the TEVAR treatment to each patient individually.

A number of studies have been conducted that provide insight into the thrombosis process post TEVAR. Computational studies have assessed aortic hemodynamics after TEVAR,^9–12^ while several anatomical studies^13–16^ have identified morphological parameters of the aorta that influence the progression of the disease. For example, the presence of additional reentry tears was found to reduce the chances of FL thrombosis. Moreover, geometrical features of the stent-graft itself (such as the total length or diameter of the device) were analyzed but did not show statistically significant correlations with FL thrombosis.

Stent-grafts are manufactured in set sizes, and given the limited sizing options, it is possible that not all patients are receiving a best fit (in terms of both diameter and length). Additionally, as the primary entry tear is usually the largest, a stent-graft may be chosen with the primary objective of covering this tear, without consideration of further distal tears. Moreover, little work has been done to assess the influence of distal tears and their locations on TEVAR outcome. Therefore, the objective of this study was to investigate how the distance between the distal end of the stent-graft and first post-stent reentry tear may influence FL thrombosis after TEVAR using computational modeling of flow and thrombus formation in patient-specific geometries.

## Materials and Methods

Three patients (P1, P2, and P3) treated with TEVAR using a Gore TAG device (Gore Medical, Flagstaff, AZ, USA) in the acute phase for uncomplicated type B aortic dissection as part of the ADSORB trial^4^ were included in this study. Formal ethical approval was not required for this retrospective study, as prior agreement was made to undertake computational modeling using anonymized images and data. Computed tomography (CT) scans were acquired within the first month post-TEVAR and annually for up to 3 years. CT scans were performed using the Brilliance 40 (Philips Healthcare, Best, the Netherlands), Lightspeed VCT (GE Healthcare, Waukesha, WI, USA), or Volume Zoom (Siemens Healthineers, Erlangen, Germany) scanner, with a voxel size range of (0.47–0.77) × (0.47–0.77) × (0.8–1.5) mm^3^ and a kVp of 120. Patient-specific geometries were reconstructed from CT scans using Mimics (version 19.0; Materialise, Leuven, Belgium) based on a semiautomated, threshold-based algorithm. Manual refinement was performed in order to separate the region of interest from the surroundings. For each reconstructed model, the false lumen volume was calculated by using the following expression:

(1) $$Vol_{FL}=\sum_{i=1}^{N}\frac{(S_{i,FL}+S_{i+1,FL})\cdot h}{2}$$

where *S_i,FL_* is the cross-sectional area of the FL for each axial slice *i, N* is the total number of axial slices, and *h* is the slice thickness. The FL was present only in the descending aorta, distal to the left subclavian artery, for all patients. Percentage changes in FL volume between the first and 1-year follow-up scans were calculated, as not all patients had follow-up imaging beyond 1 year.

**Figure 1.**
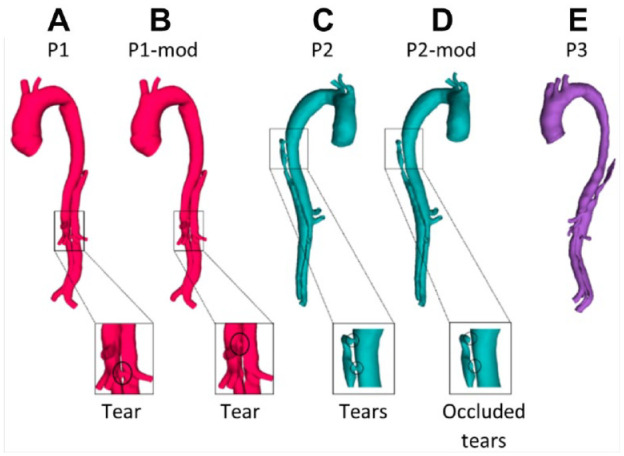
(A) P1, first postprocedure geometry; (B) P1-mod, modified geometry of P1 with the first post-stent reentry tear artificially moved proximally by 30 mm; (C) P2, first postprocedure geometry; (D) P2-mod, modified geometry of P2 with 2 proximal entry tears artificially occluded; and (E) P3, first postprocedure geometry.

In each reconstructed model, the distance between the distal end of the stent-graft and first post-stent reentry tear (SG-FRT) was measured along the centerline, which was fit using an automatic function in Mimics. To investigate the role of the SG-FRT distance, additional models were created. In the first post-TEVAR scan of P1, the FRT (originally located at the level of the left renal artery branching off the FL) was artificially moved upward by 30 mm toward the stent. In the first post-TEVAR scan of P2, two post-stent tears close to the distal end of the stent-graft were occluded to simulate the extension of the stent-graft by 30 mm. Figure 1 shows P1 and P2 and their respective modified models (P1-mod and P2-mod) alongside P3. Each model included 8 to 9 side branches: innominate artery, left common carotid artery, left subclavian artery (occluded for P1), celiac and superior mesenteric arteries, left and right renal arteries, and left and right iliac arteries.

The 5 geometrical models were imported into ICEM CFD software (Ansys Inc, Canonsburg, PA, USA) and discretized into unstructured meshes consisting of 5 to 10 million elements each. Mesh sensitivity tests were carried out for all models to ensure mesh-independent solutions. In order to focus on the influence of the SG-FRT distance on FL thrombosis, the same pulsatile inlet flow waveform^17^ was applied in all models. Since the 3-element windkessel model was recommended as the most appropriate outlet boundary condition,^18^ this was applied at each outlet with the relevant parameters taken from the literature.^17^ All flow simulations were performed using Ansys CFX (version 15.0; Ansys Inc). A fixed time step of 0.005 seconds was chosen based on previous sensitivity tests, and each model was simulated for 4 cardiac cycles to ensure a periodic solution. Results from the last cardiac cycle were used to initialize the thrombosis model.

To simulate thrombus formation and growth over time, the shear-driven thrombosis model developed and validated in the previous work of Menichini et al^11,19,20^ was implemented. A time step of 0.005 seconds was used, and simulations were run until there were no further changes in predicted thrombus patterns. The number of cardiac cycles required varied between models: 20 cycles for P1 and P1-mod, 22 for P2 and P2-mod, and 20 for P3. For P2, two intercostal arteries were present in line with the 2 upper thoracic tears, meaning that flow from the true lumen would be diverted to these arteries as well as through the 2 tears. As the intercostal arteries were too small to segment from the CT scan, they were excluded from the computational model, resulting in artificially increased flow through the tears and thus higher time-averaged wall shear stress (TAWSS). TAWSS is a key parameter on which the coagulant flux boundary condition in the thrombosis model is dependent; therefore, a modified coagulant flux boundary condition from a previous study^20^ was applied in this region for P2. This condition gives a constant coagulant production balanced by coagulant consumption dependent on time-averaged shear rates.

Predicted thrombus patterns were validated against the actual thrombus formation observed from follow-up scans. Additionally, comparisons were made between the original and modified models to assess the influence of SG-FRT distance on FL thrombosis.

## Results

### Anatomical Characteristics

Percentage changes in the FL volume between the first post-TEVAR and 1-year follow-up scans are reported in Table 1. All 3 patients had partial FL thrombosis, with P1 showing complete thrombosis in the thoracic FL but partial thrombosis in the abdominal FL. Both P2 and P3 had near complete thrombosis in the thoracic FL, with varying degrees of thrombosis in the abdominal FL. The SG-FRT distance varied across patients (Table 1), with P3 having the largest SG-FRT distance, followed by P1. P2 had a SG-FRT distance of 0 mm due to the presence of 2 tears just below the distal end of the stent-graft. The modified models had a reduced SG-FRT distance in P1-mod compared to P1 and increased SG-FRT distance in the P2-mod compared with P2.

**Table 1.** Anatomical Measurements for Each Model.

	P1	P1-mod	P2	P2-mod	P3
Thoracic false lumen volume,^a^ %	−99.9	—	−83.2	—	−87.6
Abdominal false lumen volume,^a^ %	−15.6	—	−63.4	—	−32.7
SG-FRT distance, mm	111	81	0	141	155

Abbreviation: SG-FRT, distal end of the stent-graft to first reentry tear.

a False lumen volume percentage changes were taken between the first postprocedure scan and the 1-year follow-up. Volume changes are not reported for P1-mod and P2-mod as these are artificially modified models.

### Flow Patterns and Related Parameters

**Figure 2.**
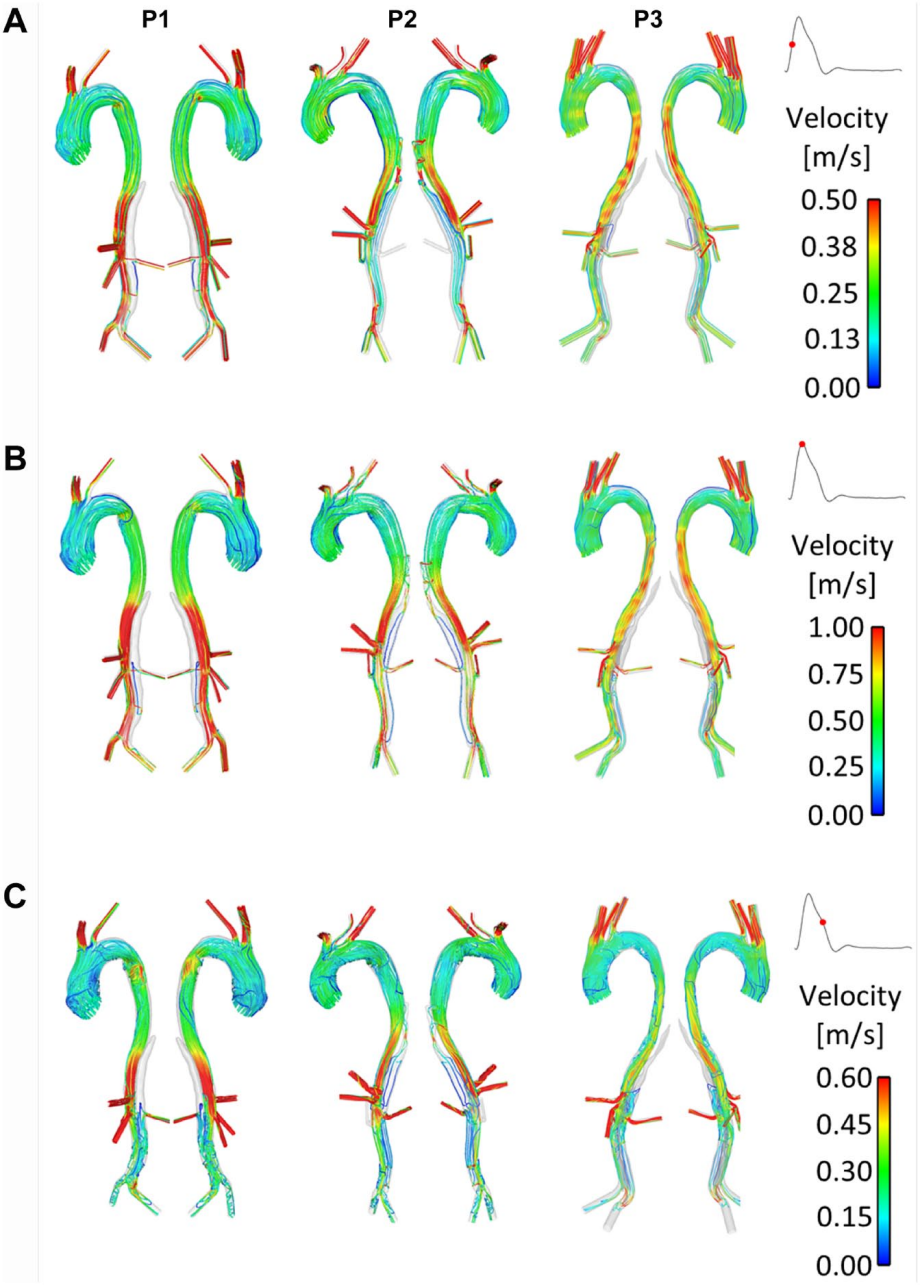
Instantaneous velocity streamlines at (A) midsystolic acceleration, (B) peak systole, and (C) midsystolic deceleration in P1, P2, and P3.

**Figure 3.**
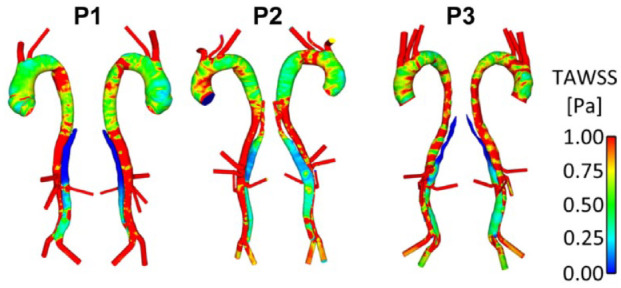
Time-averaged wall shear stress (TAWSS) distribution in the 3 patients (P1, P2, and P3).

Figures 2 and 3 show instantaneous velocity streamlines and TAWSS, respectively, before initiating the thrombosis simulation. In P1 and P3 very little flow was observed in the thoracic FL throughout the cardiac cycle. These 2 patients presented large SG-FRT distances, allowing flow to enter the distal FL, and there was no pressure gradient to drive the flow into the upper thoracic FL. This resulted in very low TAWSS in this region, as seen in Figure 3. P2, however, had higher flow and TAWSS in the thoracic FL due to proximal entry tears. In all patients, high TAWSS was observed in areas proximal to tears and branches. Disturbed flow with flow recirculation between the tears was observed in the distal FL during the deceleration phase in all 3 patients, who notably all had tears near the bifurcation, creating an outflow channel for the FL.

**Figure 4.**
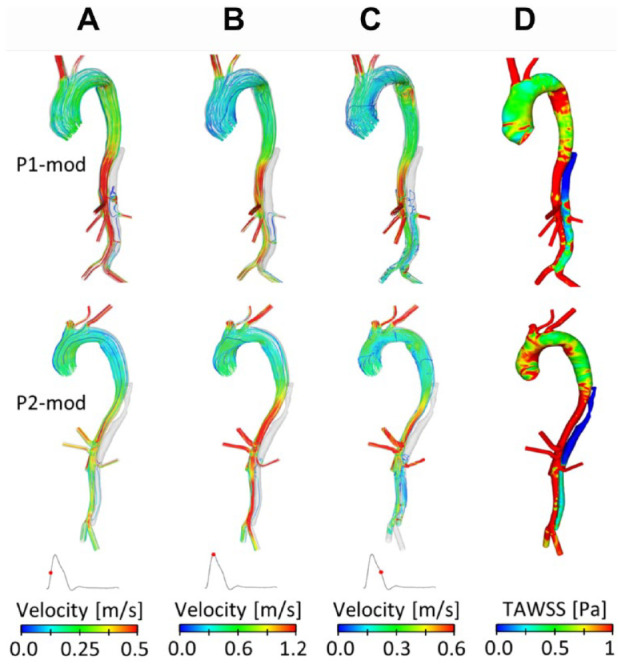
Instantaneous velocity streamlines at (A) midsystolic acceleration, (B) peak systole, and (C) midsystolic deceleration, and (D) time-averaged wall shear stress (TAWSS) for P1-mod and P2-mod.

Figure 4 shows instantaneous velocity streamlines and TAWSS for P1-mod and P2-mod. It can be clearly observed that the change of tear location in P1-mod had repercussions on FL flow and TAWSS, in particular in the region surrounding the tear. In the original model, the tear was in line with the renal arteries, so that the flow entering the FL either went straight into the left renal artery or to the abdominal FL. Therefore, almost no FL flow was observed above the renal arteries. Moving the tear upward resulted in an appreciable amount of flow in the region above the renal arteries, which circulated throughout the cardiac cycle. This resulted in an area of significantly higher TAWSS on the outer aortic wall opposite the relocated tear.

For P2-mod, covering the 2 proximal entry tears drastically altered the FL hemodynamics. There was no flow in the upper thoracic FL, resulting in very low TAWSS in this region. Additionally, the average TAWSS in the abdominal FL decreased slightly (0.52 Pa compared with 0.55 Pa in P2). The amount of flow entering the FL-perfused left renal artery was calculated, and very little change was observed between the original and modified geometries.

### Thrombus Formation in the False Lumen

**Figure 5.**
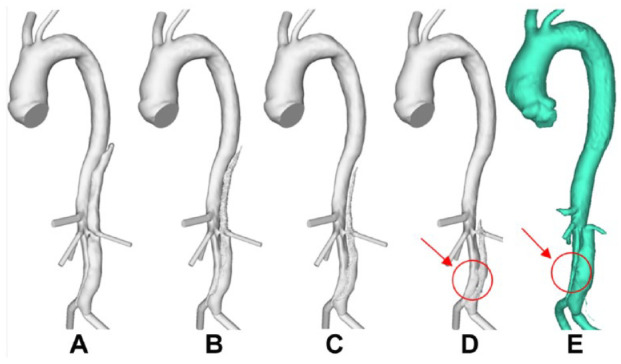
Evolution of false lumen (FL) surface in P1 following thrombus growth. (A) Reconstructed lumen surface based on the first postprocedure scan for P1 and predicted FL surface following thrombus growth at (B) 8, (C) 15, and (D) 26 seconds in comparison with (E) the reconstructed lumen surface based on follow-up scans acquired at 3 years postprocedure. This simulation shows over-prediction of thrombus growth due to the exclusion of minor branches.

**Figure 6.**
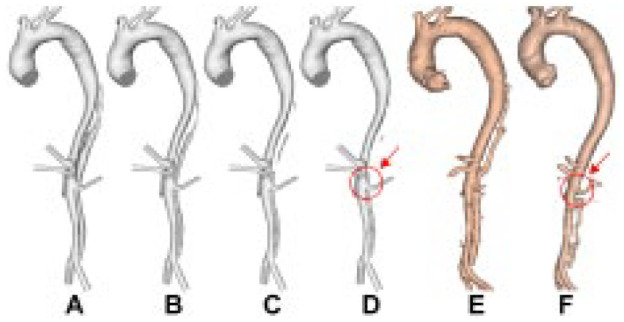
Evolution of false lumen (FL) surface in P2 following thrombus growth. (A) Reconstructed lumen surface based on the first postprocedure scan in P2 and predicted FL surface following thrombus growth at (B) 15, (C) 22, and (D) 28 seconds in comparison with the reconstructed lumen surface based on follow-up scans acquired at (E) 1 year and (F) 2 years postprocedure. This simulation shows under-prediction of thrombus growth due to the exclusion of minor branches.

**Figure 7.**
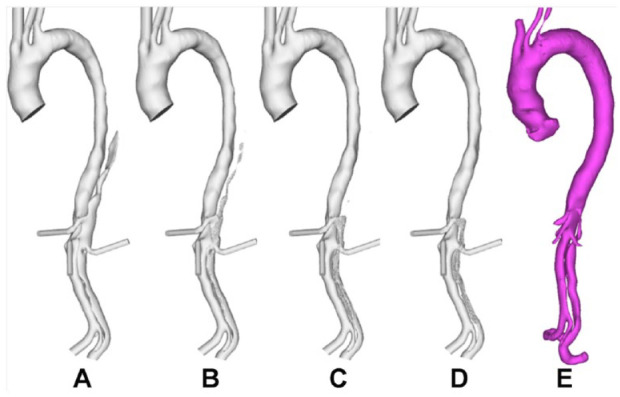
Evolution of false lumen (FL) surface in P3 following thrombus growth. (A) Reconstructed lumen surface based on the first postprocedure scan in P3 and predicted FL surface following thrombus growth at (B) 8, (C) 15, and (D) 26 seconds in comparison with (E) the reconstructed lumen surface based on follow-up scans acquired at 1 year postprocedure.

The predicted thrombus growth patterns for the original models are shown in Figures 5A-D, 6A-D, and 7A-D, along with the real geometries reconstructed from the corresponding follow-up CT scans shown in Figures 5E, 6E and F, and 7E. For all 3 patients, partial thrombosis was predicted in the FL where thrombus formation started from the top region and gradually expanded toward the first uncovered tear. A second region of partial thrombosis was also observed in-between the first and distal tears. For all patients, thrombus growth slowed down after about 20 seconds, until stopping at 26 to 28 seconds.

Comparisons with follow-up scans revealed a good overall agreement between the predicted thrombus growth and actual clinical observations. The model was able to capture the location of thrombus growth and correctly predict incomplete thrombosis within a simulated time frame of 30 seconds. Some disagreements can be observed in P1 and P2. In P1 (comparing Figure 5D and E), the model over-predicted thrombus growth in the region between the renal arteries and the aortic bifurcation. This was due to the inferior mesenteric artery and one of the intercostal arteries (not included in the computational models) branching off the FL. In P2, the region between the superior mesenteric artery and the right renal artery was predicted to remain patent, while thrombosis was observed during follow-up (highlighted in Figure 6D and F). Thrombosis of this region in the computational model was hindered by the presence of a tear at the level of the superior mesenteric artery, which allowed flow into the FL. An intercostal artery can be seen in the CT scan at the same level as the tear. Therefore, most of the flow crossing the tear would have been diverted to the branch, reducing the amount of blood circulating in this region of the FL. A very good agreement was found in P3 (comparing Figure 7D and E).

**Figure 8.**
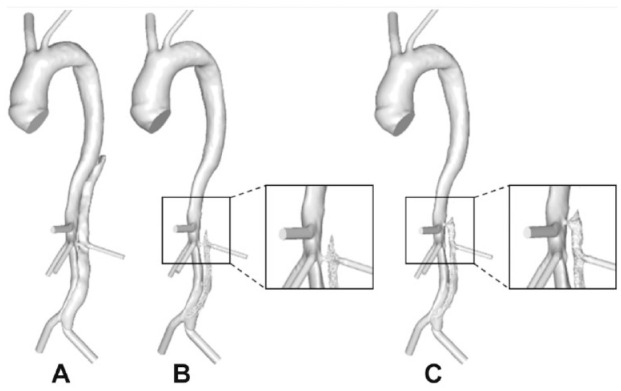
(A) Reconstructed postprocedure geometry of P1. Predicted thrombus formation for P1 in (B) the original geometry and (C) the modified geometry. The difference in predictions due to modified reentry tear position is highlighted.

**Figure 9.**
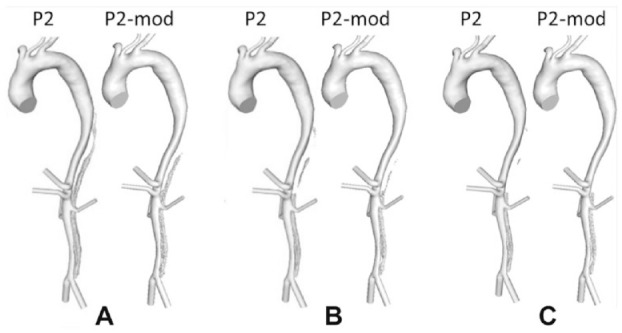
Evolution of P2 and P2-mod false lumen surface following thrombus growth compared throughout the simulation at (A) 15, (B) 22, and (C) 28 seconds.

Figure 8 shows the final predicted thrombus formation in P1 and P1-mod. It can be seen that moving the tear proximally by 30 mm resulted in reduced thrombus formation in the upper abdominal FL around the region of the left renal artery; overall, the volume of thrombus formed decreased by 9.6%. This was due to increased flow and TAWSS in this region, as seen in Figure 4. Figure 9 shows the predicted thrombus formation for P2 and P2-mod, where covering the proximal entry tears resulted in faster thrombosis of the thoracic FL as well as an increase of 4.7% in total thrombus volume. This is due to the substantially reduced thoracic TAWSS, as observed in Figure 4.

## Discussion

Although in-hospital survival rates are high for type B aortic dissection patients, the prognosis after discharge remains uncertain, with registry data suggesting a 50% mortality rate at 5 years due to aortic rupture.^21^ The key driver for aortic expansion in chronic type B dissection is the presence of blood flow within the FL, and the therapeutic goal of TEVAR is to depressurize the FL, resulting in thrombosis.

Extensive studies have been carried out trying to assess the efficacy of TEVAR against medical treatments^3–6^ and to identify parameters that can predict post-TEVAR outcomes.^13–16^ Nonetheless, it is still unclear which are the key parameters driving the progression of this disease and determining the outcome of treatments. The main aim of this study was therefore to elucidate the role of certain morphological features, FL thrombosis in particular, in determining patient outcomes post TEVAR. As the presence of patent post-stent reentry tears had been identified as a key predictor of reduced FL thrombosis,^16^ this parameter was a focus of the current study. Additionally, stent-graft design has been suggested to influence the outcome of TEVAR, either positively by promoting FL thrombosis or negatively by inducing further complications.^8^ Thus, the length of the stent-graft was taken into consideration in combination with the presence of reentry tears by studying the distance between the stent-graft and first reentry tear.

Comparison of the original patient-specific models showed substantially lower flow and TAWSS in the thoracic FL for P1 and P3 (who had a large SG-FRT distance) compared with P2, initially suggesting that the SG-FRT distance may be a key parameter in determining FL flow and thus FL thrombosis. In order to investigate this parameter while keeping all other morphological parameters constant, modifications were made to P1 and P2. The reduction of the SG-FRT distance in P1, by moving the FRT proximally by 30 mm, resulted in increased FL flow and TAWSS in the region adjacent to the tear and reduced FL thrombosis. The occurrence of a stent-graft–induced new entry tear (SINE) would also drastically decrease the SG-FRT distance. The detrimental influence of SINE on FL thrombosis and aortic remodeling has been highlighted in the literature and is in line with the results of this study showing that a decrease in SG-FRT reduces FL thrombosis.^22,23^

Increasing the SG-FRT distance in P2 by artificially extending the stent-graft 30 mm to cover the 2 adjacent reentry tears led to reduced FL flow and TAWSS, as well as faster and increased FL thrombosis. These results indicate that a large distance between the distal end of the stent-graft and the FRT would be favorable for FL thrombosis. Additionally, it was shown that occluding these tears by extension of the stent-graft had little impact on the flow to the FL-perfused left renal artery. This means a longer stent-graft could have been used for this patient without adversely affecting left renal artery perfusion. In this case the extension of the stent-graft was by only 30 mm, with 100 mm between the distal end of the stent-graft and the celiac trunk remaining uncovered. However, it has been highlighted that extended coverage of the entire thoracic aorta up to the celiac trunk may be linked to an increased risk of spinal cord ischemia.^24^ This should be considered when choosing the stent-graft length, and the desire to cover additional tears should be balanced with the increased risk of such a complication. In the case where a longer stent-graft is used to occlude additional reentry tears, reducing the stent-graft length to increase the SG-FRT distance would cause the most distal covered reentry tear to be exposed, resetting the SG-FRT to zero. Therefore, in this scenario, artificially increasing the SG-FRT distance by decreasing stent-graft length would not benefit FL thrombosis.

Following on the previous work of predicting FL thrombosis in TEVAR patients,^11^ the model complexity was increased to include major side branches. Consistency between simulated thrombus formation and follow-up CT scans further demonstrated the validity of the predictive model. Slight deviations in results were mainly attributed to the presence of minor FL-perfused branches. In regions where small branches were artificially occluded, the model overpredicted thrombus formation. This is due to the fact that the presence of FL branches creates an additional pressure drop that drives flow within the FL and hinders the deposition of platelets and the formation of thrombus. This factor has been highlighted in several anatomical studies for both TEVAR and medical management patients.^13–16^

Although much work has been done to identify the effects of changing morphological features on the presence of FL thrombosis after TEVAR, little had been done prior to this work to quantify thrombus formation and assess the direct effect of such parameters on changes in FL volume, especially looking at specific regions of the aorta. Our aim was to understand the physics behind key morphological parameters related to both the patient and the stent-graft. While there are numerous biological and morphological factors that contribute to FL thrombosis after TEVAR, the focus of this study on one specific parameter highlights the significant influence that a single variable can have on patient outcome. It is hoped that these findings will help us elucidate the dynamics driving the progression of type B aortic dissection for endovascularly treated patients, which will further help clinicians develop more effective treatment strategies for individual patients.

### Limitations

The work presented here has several limitations. First, the number of patients included was small. Dissections can present in a wide range of morphologies and analysis of a larger cohort would provide the opportunity to study morphological parameters in varied cases. Second, all patients included in the present study were treated with a GORE TAG device in the TEVAR procedure. It would be necessary to extend the study to patients treated with different devices in order to elucidate the effect of stent-graft design on the predicted outcome.

With regard to the computational model, while previous work has validated the thrombosis model for predicting clinical outcomes,^11,19,20^ and the results from this study show good agreement between model predictions and follow-up scans, there is room for further refinement. The patient-specific computational models did not include minor side branches (such as intercostal arteries), and our results showed that excluding such branches could influence the predicted thrombus formation.

Additionally, all models in this study assumed rigid wall behavior. It is known that the intimal flap is initially mobile and becomes stiffer as the dissection progresses to the chronic phase,^25^ which could affect predictions of flow and thrombus growth in dissected regions not covered by the stent-graft. Furthermore, the inflexibility of the stent-graft and its relative stiffness compared to the aortic wall may impact aortic hemodynamics and thrombus formation.

## Conclusion

Our results demonstrate that the SG-FRT distance can influence FL hemodynamics and thrombus formation, with a large SG-FRT favoring FL thrombosis. These findings could potentially help clinicians select the most appropriate treatments for individual patients and to predict which patients would be most likely to benefit from endovascular treatments. Future studies of a larger cohort of patients will be beneficial to assess the impact of SG-FRT distance on a wide range of dissection morphologies. Additionally, further studies using our methodology of modifying patient-specific geometries will facilitate similar analysis of other key morphological parameters to determine which anatomical factors play the most important role in the progression of aortic dissection.
